# Sparse ab initio x-ray transmission spectrotomography for nanoscopic compositional analysis of functional materials

**DOI:** 10.1126/sciadv.abf6971

**Published:** 2021-06-09

**Authors:** Zirui Gao, Michal Odstrcil, Sebastian Böcklein, Dennis Palagin, Mirko Holler, Dario Ferreira Sanchez, Frank Krumeich, Andreas Menzel, Marco Stampanoni, Gerhard Mestl, Jeroen Anton van Bokhoven, Manuel Guizar-Sicairos, Johannes Ihli

**Affiliations:** 1 Paul Scherrer Institut, 5232 Villigen PSI, Switzerland.; 2 ETH and University of Zürich, Institute for Biomedical Engineering, 8092 Zürich, Switzerland.; 3 Carl Zeiss SMT GmbH, 73447 Oberkochen, Germany.; 4 Clariant AG, 83052 Bruckmühl, Germany.; 5 ETH Zürich, Institute for Chemical and Bioengineering, 8093 Zürich, Switzerland.

## Abstract

The performance of functional materials is either driven or limited by nanoscopic heterogeneities distributed throughout the material’s volume. To better our understanding of these materials, we need characterization tools that allow us to determine the nature and distribution of these heterogeneities in their native geometry in 3D. Here, we introduce a method based on x-ray near-edge spectroscopy, ptychographic x-ray computed nanotomography, and sparsity techniques. The method allows the acquisition of quantitative multimodal tomograms of representative sample volumes at sub–30 nm half-period spatial resolution within practical acquisition times, which enables local structure refinements in complex geometries. To demonstrate the method’s capabilities, we investigated the transformation of vanadium phosphorus oxide catalysts with industrial use. We observe changes from the micrometer to the atomic level and the formation of a location-specific defect so far only theorized. These results led to a reevaluation of these catalysts used in the production of plastics.

## Teaser

We developed a spectro-nanotomography method that enables fast chemical characterization of functional materials.

## INTRODUCTION

Composition and structure define a material’s functionality (*1*). While we can determine and sometimes predict the relationship between structure and emergent functionality for simple single-component materials with some effort, we frequently face difficulties when dealing with structurally and compositionally more complex materials such as heterogeneous catalysts, energy storage materials, or biominerals (*2*–*4*). Here, functionality is often defined by local heterogeneities in structure and/or composition such as interfaces between two components or selected crystallographic defects, distributed in a larger volume (*5*, *6*). The distribution of these heterogeneities within frequently hierarchically structured assemblies, spanning multiple length scales, and their interaction with the local environment further guide the material’s functionality or device performance. Hence, we face the challenge to provide characterization tools that allow us to determine the nature and distribution of these heterogeneities in their native geometry in three dimensions (3D). This is to better our understanding of current materials and aid the design of next-generation materials.

X-ray absorption near-edge spectroscopy (XANES), the measurement of x-ray excitation characteristics of a chemical element in response to variation in incident energy, has become the dominant method for chemical speciation and component identification in various research fields (*7*, *8*). While initially limited to bulk analysis, the increasing importance of advanced composite materials (*3*, *4*, *6*, *9*–*11*) has led to the development of XANES imaging and, eventually, to XANES tomography (*12*–*18*), adding a structural characterization element and aiming to identify and localize local heterogeneities in a system-representative sample volume, i.e., providing the desired characterization tool. These techniques are especially of interest when aiming for nanoscale resolution to reveal features at the scale in which different chemical phases intertwine in these materials (*3*, *4*, *6*, *9*–*11*). However, current XANES tomography implementations (*12*–*18*) suffer from two particular difficulties when in pursuit of nanoscopic features in representative sample volumes: Access to local quantitative density or elemental concentrations requires effort in calibration and normalization that is often overlooked, and lengthy acquisition times (*19*). Until now, spectral tomogram synthesis involved the acquisition of one tomogram per energy to generate the hyperspectral dataset. The required number of projections per tomogram scales with the sought spatial resolution and the diameter of the sample following the Crowther criterion (*20*). Iterative reconstruction methods, such as the simultaneous algebraic reconstruction technique (SART) (*21*), were introduced to relax the number of projections while preserving the quality of the tomographic reconstruction.

Here, we introduce a novel acquisition scheme and iterative reconstruction technique that leverages the sparsity of information in a hyperspectral tomogram to relax the required number of projections further, thereby substantially reducing XANES tomogram acquisition times (*19*). Specifically, using the introduced reconstruction technique, we were able to reduce the number of projections to 11% of the Crowther criterion at no noticeable cost of spectral or spatial resolution. Such a reduction is possible, as signal variation across the spectra is heavily correlated and can be reduced to spatially localized and consistent gray-level changes; our reconstruction leverages this correlation to relax the required measurements.

Although the developed reconstruction technique is applicable to a wide range of tomography techniques, we here selected ptychographic x-ray computed tomography (PXCT) (*22*) as the vehicle of choice to provide an easier or more direct access to quantitative values (*19*). PXCT readily provides quantitative tomograms of the complex-valued refractive index distribution, i.e., phase and absorption. As a lensless imaging technique, its spatial resolution is not limited by aberrations or technical limitations in the fabrication of optics, which is a substantial challenge for x-ray wavelengths; this makes PXCT prolific in terms of signal-to-noise ratio (SNR) and with outstanding resolving power. The combination of PXCT’s high resolution and quantitativeness with our sparse reconstruction method for spectral tomography, termed sparse x-ray transmission near-edge spectrotomography (XTNES), enables the acquisition of a 3D picture of representative volumes with nanometer resolution, which reconstructs into quantitative values of electron density, absorption, elemental concentration, and oxidation state. This ultimately allows a local, quantitative characterization of structure, chemical composition, and coordination geometry.

In this first application, we examined a pristine and industrially used vanadium phosphorus oxide (VPO) catalyst. These oxides are used to catalyze the selective oxidation of *n*-butane (C_4_H_10_) to maleic anhydride (MA) (C_4_H_2_O_3_). MA is a precursor in the production of plastics, with a steadily increasing production quantity of currently 2 million tons per year (*23*, *24*). In consideration of the reaction by-products, carbon monoxide and carbon dioxide, a financial and environmental incentive is present to increase the productivity of these catalysts. State-of-the-art catalysts are a composite of hierarchical porosity, and one of the factors that hinder catalyst improvement is that the composition and spatial distribution of its vanadium phosphate phases (table S1) are not precisely known. Another factor is that, during reactor operation, the catalyst undergoes a series of structural and compositional changes that culminate in a gradual loss of catalyst productivity. Because of the compositional uncertainty, the aforementioned changes are yet to be fully understood, leading to an active discussion regarding the best catalyst design, the most desirable active phase, and the nature of active sites in general (*25*–*29*). Historically, V═O bonds or V^5+/^V^4+^ redox pairs on the catalyst’s surface are considered to be the active sites in the initial hydrogen transfer reaction to activate *n*-butane on the catalyst surface. The former, i.e., the cleaving of alkane C─H bonds, is suggested to be the rate-limiting step (*24*, *26*, *28*–*31*). More recently, P═O bonds were theoretically suggested to play an equally important role (*32*, *33*). Naturally, materials of increased structural disorder, exhibiting more of these bonds at their surfaces, find use in industrial VPO catalysts (*34*, *35*), for example, defect-rich nanoparticles and amorphous phases. Please see the Supplementary Materials and fig. S1 for further details regarding VPO catalysts and their industrial use.

The XTNES tomography measurements presented here provide answers to some of these uncertainties, explicitly those surrounding catalyst structure, composition, active sites, and productivity. The multimodal hyperspectral tomograms revealed a structural and chemical transformation following 4 years of industrial reactor utilization, that is, from a mesoporous catalyst of high surface area composed of a series of amorphous and nanocrystalline vanadium phosphate phases to a macroporous catalyst composed of micrometer-sized and defect-rich vanadyl pyrophosphate crystals. When evaluated against catalytic performance, this transformation directly implies the used catalyst to be more productive on a surface area–specific basis than the pristine and equilibrated catalyst. By using the quantitativeness of XTNES to perform local structure optimizations on vanadyl pyrophosphate crystals that were in contact with the reactive medium, we were able to correlate this increased productivity of the used catalyst to vanadyl defects, which create unsaturated P═O bonds that are accessible from {200}-terminated facets. Hence, catalysts of highest activity might not be derivable from amorphous surface deposits enriched in V^5+^ or the interaction of nanometer-sized V^5+^ and V^4+^ components as previously targeted but rather through defect engineering of highly crystalline vanadyl pyrophosphate crystals (*30*, *31*). These observations currently aid the design of improved VPO catalysts and hopefully highlight the prowess of XTNES tomography including local defect and potential active site characterization.

## RESULTS

### X-ray transmission near-edge spectrotomography

**Fig. 1:**
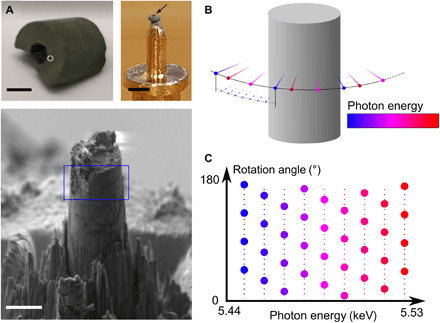
Illustration of sparse XTNES acquisition. (**A**) Examined VPO catalyst pellet as retrieved from the reactor (top left). Preshaped sample mounted on a tomography pin (top right, black arrow) and focused ion beam milled sample cylinder (bottom). Scale bars are 5 mm, 1 mm and 10 μm. The white circle indicates the region from which the sample cylinder was extracted. The blue rectangle indicates roughly the field of view during XTNES tomogram acquisition. Photo credit: Zirui Gao, PSI, ETHZ. (**B**) Spectral tomograms were assembled by acquiring a series of angularly sparse ptychographic tomograms across the vanadium *K*-edge. (**C**) Graphical illustration of the acquisition scheme of tomography angular orientations versus x-ray photon energy. For each energy, the Crowther criterion is indicated by small dots, while larger dots indicate angularly sparse measured projections. At each energy, an offset to the angle based on a golden ratio is added to each projection to maximize the available information diversity.

Clariant AG provided the pristine and used VPO catalyst bodies, i.e. pellets (*36*). The latter were sourced from an industrial fixed-bed reactor after 4 years of known operation history. The reactor coolant temperature was gradually increased from 400° to ~420°C following the first year of operation to ensure steady reactor performance. From performance profiles (fig. S2), we can estimate that a catalyst equilibrium state and structure was reached after ~1.5 years of operation. Bulk examination revealed the used catalyst to exhibit a 70% decrease in specific surface area and a ~10% reduction in productivity after 4 years of operation (table S2). As shown in Fig. 1A, samples intended for tomographic examination were extracted from the central region of randomly selected catalyst pellets and shaped into cylinders roughly 12 μm in diameter.

High-resolution tomographic projections (fig. S3) were acquired at 60 x-ray photon energies across the vanadium *K*-edge, between 5.443 and 5.530 keV. For a target spatial resolution of 25 nm, a conventional spectrotomography measurement using analytical reconstruction technique, i.e., satisfying the Crowther sampling criteria, would require the acquisition of 628 projections at each energy. Following our sparse sampling approach, we measured, at each energy, only 68 projections, resulting in a reduction in acquisition time per XTNES tomogram from more than 1 week to less than 20 hours. To retain information from a high diversity of incident angles, which aids the sparse synthesis, we further introduced a unique offset to the first projection angle of each single-energy tomogram (*37*). Figure 1 (B and C) illustrates the acquisition strategy. Similar angle interleaved acquisition strategies found previous application within the field of time-resolved x-ray tomography (*37*, *38*).

At these photon energies, the phase of the complex-valued projections provides a better SNR and spatial resolution compared to the amplitude. Therefore, high-resolution processing was conducted using the phase, while the amplitude or absorption signal was used as a low-resolution reference.

The reconstruction of the sparse XTNES tomograms was carried out using a novel iterative algorithm based on principal components analysis (PCA) (*39*) and SART (*21*). The reconstruction process involves a noncentered PCA decomposition of a downsized hyperspectral tomogram to extract a set of spectral modes with their corresponding component tomograms to be used as initial guess. The initial guess is then iteratively refined using all the high-resolution projections at all energies. This process results in a set of spectral modes and their corresponding tomograms at the highest resolution, from which we can calculate the XTNES tomogram. The half-period spatial resolution of the obtained XTNES tomograms was determined using 4D Fourier shell correlation and found to be limited by the voxel size, here 26.50 nm. The corresponding full-period resolution is 53.0 nm. Please see Materials and Methods for a detailed explanation of the reconstruction procedure and fig. S4 for details regarding resolution estimates. Furthermore, the reader is referred to fig. S5, which provides a comparison of different analytical and iterative tomogram reconstruction methods to showcase the benefit of the introduced sparse spectral tomogram reconstruction method.

**Fig. 2:**
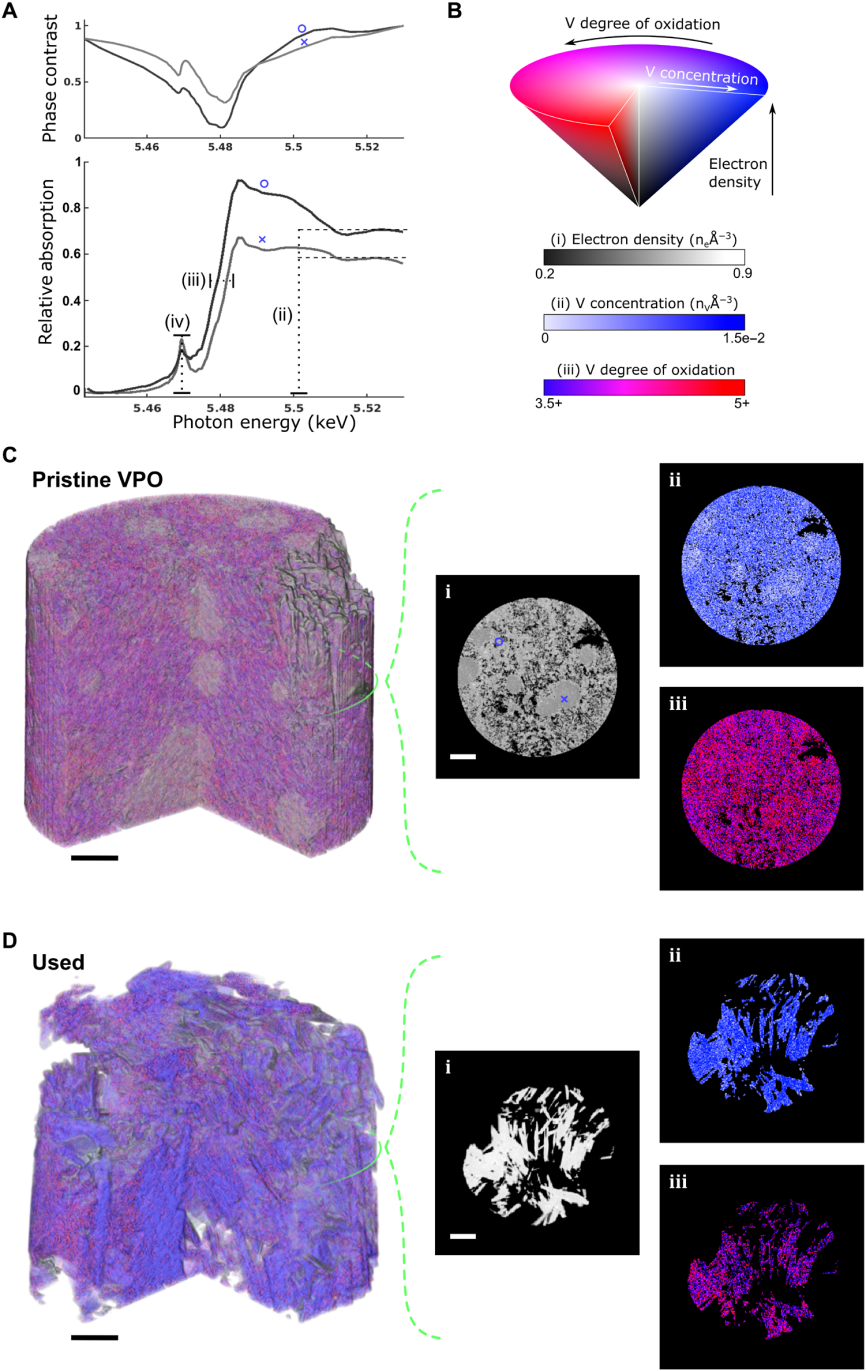
Local vanadium *K*-edge spectra and XTNES tomograms of industrial VPO catalysts. (**A**) Example of two voxel-level phase and KKT-obtained absorption spectra. From these spectra, we obtain quantitative values for (i) the electron density, (ii) vanadium concentration, (iii) vanadium oxidation state, and (iv) pre-peak intensity. (**B**) Hue saturation value 3D color map used for the combined visualization of electron density, vanadium concentration, and vanadium oxidation state. (**C**) 3D volume rendering of the pristine catalyst based on the color map presented in (B) and axial virtual slices taken from the middle of the catalyst sample highlighting the individual quantities. (**D**) 3D volume rendering and virtual slices of the used catalyst based on the color map in (B). Scale bars, 2 μm. The positions of the voxels discussed in (A) are marked in (C) using a cross and a circle, respectively.

The phase and absorption spectra of two voxels extracted from the pristine catalyst are shown in Fig. 2A. The provided absorption spectra were obtained using a Kramers-Kronig transformation (KKT) of the phase spectra (*40*). Given that this is the method’s first application, we transformed the phase signal to absorption via a KKT to allow an easier analysis and validation of the reconstructed spectra owing to the established analysis routines for XANES spectroscopy and the availability of reference spectra (*41*). For example, a comparison of one of the provided voxel-level absorption spectra with spectra from the literature, as shown in fig. S6, confirms that the voxel consists mainly of vanadyl pyrophosphate. The typical approach for the analysis of XANES spectra, as well as XANES tomography, is to compare the measured spectra to those of a list of reference components. A particular match with one, or a linear combination of multiple reference spectra, allows the identification of a single or fractional quantification of multiple components (*7*, *13*, *42*). The quantitativeness of ptychography-based XTNES tomography affords us with a different analysis approach to reach the same goal. This approach is based on the extraction of a set of key quantities directly from the acquired phase or absorption spectra of each voxel. The quantities extracted in the present case include (i) local electron densities obtained from the phase signal away from the resonant edge, (ii) local vanadium concentrations retrieved from the edge jump magnitude, (iii) local vanadium oxidation states determined by the position of the absorption edge, and (iv) local vanadium coordination geometry inferred from pre-edge peak intensity variations. Please see Fig. 2A for a graphical illustration and Materials and Methods for further details about how these quantities are obtained. Instead of a comparison of spectra, we compare the extracted set of quantities with those of a list of possible, or considered, reference components (shown in table S1). If a match of the measured set with those of a single reference component is not possible, a linear combination of multiple reference components can be explored to best describe the measured set of quantities. While the typical spectrum comparison–based analysis is reliant on the availability of reference spectra or reference materials from which spectra can be collected, the quantity-based analysis is largely free of this constraint. In this study, a selected number of reference spectra of components with known oxidation state are used to convert measured edge energies to oxidation state. However, the quantities used here for component identification are also readily available in the literature for a vast amount of materials. This makes the presented approach well suited for the examination of materials where reference spectra are hard to come by, as is often the case for industrial materials. For cases in which reference spectra are available, this approach could, of course, leverage this extra information to add additional constrains for a refined material characterization.

### XTNES tomograms of pristine and deactivated VPO catalysts

Figure 2 (B to D) shows compound volume renderings of the tomogram extracted quantities (i), (ii), and (iii) for both the pristine and used VPO catalyst together with orthoslices of the individual quantities. Please refer to movies S1 and S2 for an animated representation. Associated histograms and correlations of these quantities are shown in fig. S7.

It is evident from a structural comparison of the electron density tomograms, i.e., the outcome of a conventional PXCT measurement, that the catalyst undergoes a structural reorganization during reactor operation. The catalyst’s nanoporosity and fine structure are lost, leaving behind a mesoporous structure composed of micrometer-sized domains. Accordingly, a decrease in surface area and widening of the pore network is registered, as shown in fig. S8 and table S2. An increase in average electron density (±σ) from 0.66 (± 0.007) to 0.86 (± 0.006) n_e_Å^−3^ further suggests that the observed changes in structure are a product of phase transformation processes. Last, on the basis of electron density distribution, as shown in Fig. 2 (C and D) and fig. S7, there appears to be little compositional variance within the pristine and used catalyst. While this may appear to conflict with the reported ill-defined nature of VPO catalysts (*25*–*29*), in reality, we find numerous industrial VPO catalyst components to have comparable electron densities. The situation is similar for vanadium concentration and vanadium oxidation state (table S1). This renders schemes based on a single value for component identification insufficient. For example, an electron density of 0.86 n_e_Å^−3^ as dominantly observed in the used catalyst could equally be interpreted, among others, as nanocrystalline (VO)_2_P_2_O_7_, V(PO_3_)_3_, and VO(HPO_4_)·0.5H_2_O, all known to be possible catalyst components (*26*, *28*, *34*, *35*).

An identification of components becomes possible, when electron density, vanadium concentration, and vanadium oxidation state are considered together, as demonstrated in fig. S7. On the basis of this correlative identification approach, we identified three distinct components in the pristine catalyst. Identification was limited to materials reported in the literature to be present in VPO catalysts, as shown in table S1 (*26*, *28*, *34*, *35*). These are amorphous vanadium-rich metaphosphate, nanocrystalline vanadyl pyrophosphate [(VO)_2_P_2_O_7_)], and vanadium hydrogen phosphate [~(VO)PO_4_·2H_2_O]. While the latter is present in the form of isolated, micrometer-sized objects, the former two, which account for the majority of the sample volume, are found in nano-sized domains. Notably, domains of these two components are spatially intertwined, with the minor metaphosphate phase preferentially facing the pore space or reaction environment, thereby increasing the vanadium concentration and oxidation state at the catalyst surface. The catalyst composition changes with reactor operation. In the used catalyst, we identified only two components, namely, small islands of VOPO_4_ (<10 volume %) and a dominantly defect-rich vanadyl pyrophosphate. The latter appears throughout the entire catalyst in micrometer-sized domains that exhibit a variance in vanadium concentration and oxidation state, which are known to occur in solid-state transformation processes. See figs. S9 to S12 for electron microscopy, x-ray fluorescence, and x-ray diffraction measurements. Each measurement confirms a selected general aspect of the XTNES tomogram analysis, such as changes in pore and fine structure, the loss/absence of amorphous material, variation in vanadium concentration, and the general presence of identified components.

**Fig. 3:**
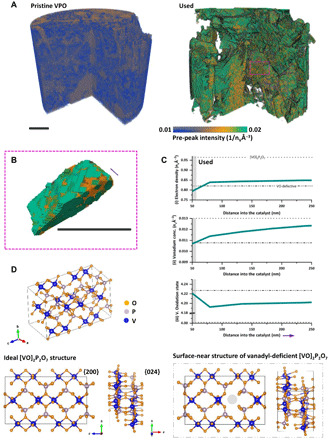
Microstructural analysis, local structure optimization, and defect Identification in vanadium phosphorus oxide catalysts. (**A**) Pre-peak intensity volume renderings, shown as (iv) in Fig. 2A, of the pristine and used VPO catalyst. (**B**) Volume rendering of the pre-peak intensity of a single vanadyl pyrophosphate [(VO)_2_P_2_O_7_] grain found in the used VPO catalyst. Changes in pre-peak intensity are displayed in the form of a gradient color map ranging from blue to green. Scale bars, 2 μm. (**C**) 3D distance maps of XTNES extracted quantities—(i), (ii), and (iii)—from the pore space into the used VPO catalyst. Magnitudes encountered within the first 80 nm of the material are shown in gray, which were used as boundary conditions for ground-state total energy calculations of the crystal structure of vanadyl pyrophosphate in contact with the reactive medium, thereby found to be vanadyl deficient (VO). The gray dotted lines show reference values of both the ideal and defective structure. (**D**) Ideal crystal structure of vanadyl pyrophosphate including {200} and {024} surface projections as well as vanadyl defective projections of the calculated catalyst structure in contact with the reactive medium (gray box).

Intensity variations of the vanadium pre-edge peak (iv) are discussed separately and shown in Fig. 3 (A and B). These variations provide a first glance at the microstructure of the catalysts, e.g., allowing the detection of crystalline and amorphous domains (*43*). This is made possible by the combination of using a linearly polarized illumination and the net anisotropy of most crystalline materials (*44*, *45*). Notably, it is the spatial extent of regions with increased pre-peak intensities that reaffirm the loss of amorphous domains and the catalyst’s nanocrystalline structure during reactor use. In the used catalyst, we find vanadyl pyrophosphate crystals with a coherent domain size of multiple micrometers, which suggests the presence of chemically more defined surfaces than were available in the pristine catalyst. See figs. S12 and S13 for a confirmation by powder x-ray diffraction and electron microscopy. On the basis of the Bragg peak intensity ratio of the vanadyl pyrophosphate reflections, these surfaces, which are available for selective oxidation, are present in the form of {200}-terminated facets and, with progressive use, increasingly {024}-terminated facets.

To obtain a better understanding of the defect-rich crystal structure of these surfaces, which drive the catalytic conversion process, we calculated the average electron density, vanadium concentration, and vanadium oxidation state of vanadyl pyrophosphate in direct contact with the reactive medium, as shown in Fig. 3C. These values were combined with ratiometric phosphorus/vanadium (P/V) measurements (fig. S11) to locally specify the vanadyl pyrophosphate crystal structure by means of ground-state total energy calculations. See the Supplementary Materials for details. Figure 3D presents a comparison of the ideal vanadyl pyrophosphate crystal structure and the calculated structure of the outermost 80 nm of the used catalyst. The loss of a single vanadyl group per unit cell following reactor use is visible. The resulting vacancy is selectively accessible in the {200} direction, thereby creating unsaturated P═O bonds on the surface of these facets and increasing the oxidation state in neighboring vanadium atoms. Unsaturated P═O bonds on the {200} surfaces of vanadyl pyrophosphate have so far only been theorized to exist and were recently suggested to be active sites in the conversion of *n*-butane to MA (*32*, *33*).

## DISCUSSION

As losses in specific surface area (70%) stand in no relation to the reduction in catalyst productivity (~10%), it is hard to argue that the catalyst performs worse after reactor use. Far from it, assuming an equal density of active sites in the pristine and used catalyst, the used catalyst performs astonishingly well on a surface area–specific scale even after 4 years of reactor operation (fig. S2). This poses the question as to why and how to leverage this information to design improved VPO catalysts or increase the lifetime of existing catalysts.

Spectral nanotomography measurements provide a first answer to this question. The detected loss of amorphous vanadium-rich metaphosphate and the increasing absence of secondary vanadium phosphate phases with progressive reactor operation, suggest both features are of minor importance regarding catalyst productivity, which is unexpected given their previous consideration as active site carriers (*30*, *31*). Rather, catalyst productivity in the used catalyst appears to derive from a increased density and availability of active sites per unit area on the surface of vanadyl-deficient and crystalline vanadyl pyrophosphate grains. In addition to the previously suggested V─O bond on {200}-terminated facets, the here identified unsaturated P═O bonds (*32*) appear to contribute to the catalyst’s productivity. The latter has more uncompensated negative charge and therefore a higher affinity to extract the hydrogen atom (H) from the *n*-butane C─H bond (see the Supplementary Materials) (*33*). On the basis of these results, rather than focusing on the purposeful introduction and maintenance of amorphous vanadium-rich metaphosphate and hence V^5+^-rich phases on the catalyst surface through phosphate dosing (*23*), current catalyst design studies of industrial collaborators for improving MA yield and selectivity are targeting the production of catalysts with narrower pore space and are composed of vanadyl-deficient vanadyl pyrophosphate. Structural and compositional observations and resulting conclusions were only possible through the examination of catalysts extracted from an industrial reactor operated under low-phosphate dosing conditions [<1 parts per million by volume (ppmv)] and following long-term use, which allows us to probe an evolved catalyst structure that emerges only after years of use in an industrial setting (*30*).

In summary, we developed a novel acquisition and reconstruction procedure for angularly sparsely sampled spectrotomograms that can be used by a wide variety of imaging techniques. Application within the framework of PXCT allowed a structural, compositional, and chemically quantitative characterization of a functional material at a representative sample volume with a half-period spatial resolution of sub–30 nm or full-period resolution of sub–60 nm, providing sufficient constrains to perform ab initio structure refinements in complex geometries on the voxel level. Furthermore, the method allows quantitative spectro-nanotomography with a much reduced acquisition time and dose. This combination satisfies an existing demand of the research community (*46*) and lessens a number of difficulties in spectral tomography, such as spatial resolution limitations imposed by imaging optics. Furthermore, this combination provides a more direct access to quantitative information, e.g., electron density and elemental concentration, and reduces the acquisition time of near-edge spectral tomograms. This method effectively puts in situ studies targeting fundamental electrochemical and catalytic processes within reach.

Moreover, the increased brightness and coherent flux of emerging fourth generation synchrotrons could lead to even further increased spatial resolution and faster acquisition. These developments alongside the presented method will open the door for nanoscale extended x-ray absorption fine structure (EXAFS) (*8*, *47*) and dedicated x-ray polarization (*43*) tomography studies.

## MATERIALS AND METHODS

### Materials

Pristine and used industrial VPO catalyst pellets, roughly 5 mm in diameter, manufactured under identical conditions were provided by Clariant AG. The used catalyst pellets were sourced from an industrial fixed-bed reactor after 4 years of operation, i.e., after an overall time on steam of 38,000 hours. Specifically, they were extracted from a catalyst bed, operated in upward gas flow configuration, and were at near constant salt bath or reactor temperature. The catalyst bed housing reactor tubes had a dimension of 21 mm by 3700 mm (width by height). The hydrocarbon inlet concentration was ~1.8 mole percent (mol %), water was added to the feed steam at a concentration of ~2 mol % throughout, and phosphates were added at a concentration of <1 ppmv. The salt bath temperature was held at ~400°C until 16,000 hours of operation before gradually increasing the temperature to 420°C to ensure a steady conversion rate in the face of catalyst deactivation (see fig. S2). A gradual loss in MA yield and catalyst selectivity is observed up to ~19,000 hours of catalyst use, or 2 years of operation. The latter indicates a catalyst structure that is still evolving up to this time point. One randomly selected pellet from each of the pristine and used catalyst population was subjected to spectrotomographic examination. Samples were stored in environment-isolated containers as much as possible before examination. The industrial VPO catalysts themselves are porous bulk-type vanadium phosphate catalysts, and hence, they are predominantly composed of vanadyl pyrophosphate, (VO)_2_P_2_O_7_, and, to a lesser extent, various vanadium phosphate phases, VO*_x_*PO_4_(H_2_O)*_n_* (*23*). Furthermore, catalysts are sparsely decorated with bismuth oxide nanoparticles (figs. S9 and S12), which are added to increase catalytic activity and selectivity toward MA. Chemical composition and selected properties of both sample populations are listed in table S2.

### Sample preparation

A pristine and used VPO catalyst pellet was first mechanically fractured, after which a central piece from each pellet was mounted on a tomography pin. The mounted samples were then preshaped using a micro-lathe (*48*) to a diameter of 100 μm before being reduced to a final diameter between 10 and 12 μm using focused ion beam milling.

### Data acquisition

XTNES tomography experiments were carried out at the coherent small-angle x-ray scattering (cSAXS) beamline of the Swiss Light Source, Paul Scherrer Institut, Switzerland. The photon energy was selected using a double-crystal Si(111) monochromator. The horizontal aperture of slits, located 22 m upstream of the sample, was set to 20 μm in width to create a horizontal virtual source point that coherently illuminated a Fresnel zone plate 220 μm in diameter and with an outermost zone width of 60 nm (*49*). The Fresnel zone plate was designed with locally displaced zones to improve imaging quality and phase accuracy (*50*). Coherent diffraction patterns were acquired using an in-vacuum Eiger 1.5M area detector, with a 75-μm pixel size, placed 5.23 m downstream of the sample inside a flight tube under vacuum. For the PXCT measurements, we used the nanotomography instrument described by Holler *et al*. (*51*). This instrument incorporates interferometrically controlled 3D stages for precise nanopositioning with an accuracy reaching 10 nm, aiming to minimize motion- or geometry-induced errors that have a negative impact on the achievable spatial resolution. Each ptychographic scan, or projection, covered a field of view of ~16 μm × 10 μm (horizontal × vertical) and consisted of ~160 scanning positions with an average step size of 1 μm. Positions followed a Fermat spiral scanning pattern (*52*). The exposure time per point was 0.1 s.

Sparse XTNES tomograms were collected by acquiring a series of sparse tomograms at different photon energies across the vanadium *K*-edge. Each sparse tomogram covered the full tomographic angular range of 180°. Measurements were collected with an energy step of 0.5 eV across the vanadium *K*-edge (5.465 to 5.487 keV) and a coarser step of 5 eV in the pre-edge (5.443 to 5.465 keV) and post-edge (5.487 to 5.540 keV) regions. In total, 60 sparse tomograms were acquired, each with 11% of the Crowther criterion angular sampling (*20*). To maximize the information content for the reconstruction of a sparse XTNES tomogram, each sparse tomogram had a different starting angle given by the golden ratio sequence (*53*) but only computed in the range between zero and one angular step size. Angle interleaved acquisition strategies have found previous application within the field of time-resolved tomography (*37*, *38*).

### Ptychographic image reconstructions

For each ptychographic image reconstruction, i.e., each tomographic projection, a region of 600 pixels × 600 pixels of the detector was used, resulting in an image pixel size of 26.10 to 26.52 nm, depending on the illumination energy. Reconstructions were obtained with 200 iterations of the difference map algorithm (*54*) followed by 300 iterations of maximum likelihood refinement (*55*), using the PtychoShelves package (*56*). The reconstruction pixel size can be, in theory, adjusted by changing the region of the detector depending on the incident x-ray energy; however, in our case, this was not possible due to the small energy step size of 0.5 eV.

### Processing of ptychographic projections

Because of the superior spatial resolution and improved SNR of the phase signal in the hard x-ray regime, compared to absorption, high-resolution XTNES tomograms were reconstructed from the acquired phase signal, while the absorption was used only as a low-resolution reference (*57*). Changes in the real part of the scattering factor, which are associated with the phase, across the vanadium *K*-edge are nearly twice as large when compared to changes in the imaginary part of the scattering factor, which is associated with the absorption.

As the pixel size of projections depends on the x-ray photon energy, we resized all complex-valued ptychographic projections to a common pixel size of 26.52 nm using an interpolation based on fast fractional Fourier transform (*58*, *59*). Next, the phase of the reconstructed 2D projections was extracted by post-processing alignment and removal of constant and linear phase components, and the projections were aligned using a tomographic consistency approach (*59*).

### XTNES tomogram reconstruction

To reconstruct a XTNES tomogram from an angularly sparse, i.e., angularly undersampled, set of projections, a new iterative algorithm, based on a combination of PCA (*39*) and SART (*21*), was developed. The aim is to reconstruct a set of spectral modes *w_k_*(*E*), where *k* = 1, 2, 3, … is the mode number, and their corresponding component tomograms *X_k_*(**r**). The hyperspectral real part of the refractive index δ, which is associated with phase contrast, is then given by

$$\delta (r,E)=\sum_{k}X_{k}(r)*w_{k}(E)$$ (1)

First, to obtain a starting guess for reconstruction of *X*_1_(**r**), the measured projections at each energy and rotation angle, namely, $\left\{P_{\theta ,E}(r_{2D})\right\}$, are spatially down-sampled by a factor 16, and low-resolution tomographic reconstructions are obtained for each energy. At this resolution, the Crowther criterion is satisfied, so a conventional tomographic reconstruction per energy can be performed. We then perform a noncentered PCA on the reconstructed hyperspectral tomograms and take the first principal coefficient, namely, *w*_1_(*E*), and the corresponding 3D spatially resolved component tomogram. The tomogram was then interpolated to the original voxel size with Fourier transform–based interpolation; using this as the starting guess of *X*_1_(**r**) accelerates the convergence, while using zeros as a starting guess is also possible.

The refinement is carried out with an iterative algorithm, similar to SART, using the high-resolution projections. For each iteration, the components are updated by

$$∆X_{1}(r)=\tau *\text{FBP}\{\sum_{E}((P_{\theta ,E}(r_{2D})-{\hat{P}}_{\theta ,E}(r_{2D}))*w_{1}(E))\}$$ (2)

where τ denotes the update ratio, *P*_θ, *E*_ denotes the measured projection at rotation angle θ and energy *E*, ${\hat{P}}_{\theta ,E}$ denotes the projection computed from the current estimate, and FBP{} denotes filtered back-projection over all rotation angles and energies (*60*). The ratio τ is calculated as

$$\tau =\alpha *\frac{N_{E}}{\sum_{E}{\left[w_{1}(E)\right]}^{2}}$$ (3)

where α = 0.2 is the relaxation constant and *N*_E_ denotes the number of energies. ${\hat{P}}_{\theta ,E}$ is computed by

$${\hat{P}}_{\theta ,E}=P_{\theta }\{X_{1}(r)*w_{1}(E)\}$$ (4)

where 𝒫_θ_ denotes a tomographic projection at rotation angle θ. Note that SART normally uses a back-projection, but here, we use FBP because it showed faster convergence. This update is applied until convergence, which we defined as less than 0.1% improvement in the error criteria, which is defined as

$$ℇ=\text{RMS}\{(P_{\theta ,E}(r_{2D})-{\hat{P}}_{\theta ,E}(r_{2D}))*w_{1}(E)\}$$ (5)

where RMS denotes root mean square calculation. Notice that by reducing the error with respect to the high-resolution projections, the update step refines the resolution of the initial low-resolution guess. Once convergence is reached for *X*_1_(**r**), we proceed with refinement of the next modes. First, we remove all contributions of the first component from the projections by

$$P\prime_{\theta ,E}(r_{2D})=P_{\theta ,E}(r_{2D})-P_{\theta }\{X_{1}(r)*w_{1}(E)\}$$ (6)

Now, taking {*P*′_θ, *E*_(**r**_2*D*_)} as the new data, we down-sample again and compute a low-resolution spectral tomogram from them. The whole procedure above is then repeated to get *w*_2_(*E*) and *X*_2_(**r**). Because of the orthogonality of coefficients in PCA decomposition, the reconstruction of the latter does not interfere with previous modes. We then repeat the procedure until the fourth component tomogram *X*_4_(**r**) is reconstructed, which was, in these cases, the last component to show signal above the noise. Note that the total number of acquired projections for the pristine catalyst is 4677, which is approximately eight times those required for a single tomogram sampled following the Crowther criteria, and similar for the used catalyst. From an information content view, our measurement justifies recovery of four components.

After this process, we obtain a set of spectral modes and the corresponding component tomograms at the finest resolution. Last, the hyperspectral tomograms can be calculated using Eq. 1. This reconstruction approach leverages sparsity on the data ab initio by only allowing a finite number of orthogonal spectral modes in the reconstruction, following the logic that only a finite number of chemical variations of the resonant element(s) are in occurrence. This is unlike the methods that use spatial regularization or total variation, i.e., our approach relies on favoring neither smooth nor spatially sparse or piecewise continuous solutions.

Note that, when inspecting reconstructions, we observed that the second spectral mode was picking up small and reversible sample motions/deformations that correlated with the degree of x-ray absorption by the probed chemical element. While this observation is interesting in its own right and the topic of a future manuscript, for purposes of chemical analysis, we here discarded the use of that mode.

### Extraction of chemical information from the XTNES tomogram

Following the determination of pore volume and catalyst-associated volume by means of threshold segmentation, several spectral features were calculated for each catalyst-associated voxel (see Fig. 2A). To allow the calculation of these features, which are at times extracted from relatively small changes in the spectrum and as such susceptible to noise, we first applied a 2 × 2 × 2 binning to the reconstruction to a voxel size of 53 nm. Voxel-level phase spectra were next converted to absorption spectra using a KKT (*41*). See the “Kramers-Kronig transformation (KKT)” section in the Supplementary Materials for details. The resulting voxel-level XANES were then corrected by removing the native energy dependence of the attenuation coefficient through linear regression of the pre- and post-edge region, i.e., 5.443 to 5.465 keV and 5.495 to 5.540 keV, respectively (*16*). This correction is needed to obtain quantitative vanadium concentrations and pre-edge intensities as well as to determine the vanadium *K*-edge position following the method of Koningsberger and Prins (*61*). The vanadium concentration values were refined using corresponding features in the phase signal. Voxels with a vanadium concentration of less than 10^−3^ n_v_Å^−3^ were removed from further analysis. Vanadium *K*-edge positions, i.e., resonant edge energies, were determined using the method of Koningsberger and Prins (*61*) and then converted to the vanadium oxidation state. This conversion was achieved through linear interpolation using the known edge energies of V(PO_3_)_3_ and (VO)PO_4_ as lower and upper bound. V(PO_3_)_3_ and (VO)PO_4_ were selected as reference components due to their known vanadium oxidation state V3^+^ and V5^+^ and known possible components present within VPO catalysts (see table S1). This conversion from edge energy to oxidation state resulted in a couple of voxels with an apparent oxidation state of greater than V^5+^ as can be seen in the corresponding histograms (fig. S7). These unrealistic values are a result of reference point selection and edge position measurement uncertainty in selected voxels, i.e., those where spectrum normalization was difficult or failed. The general reliability of the applied phase-to-absorption KKT was validated by comparing the resulting XANES spectrum with the XANES spectrum extracted from the absorption component of ptychography images and with a XANES spectrum of the same VPO catalyst acquired using a dedicated x-ray absorption spectromicroscopy setup (fig. S14).

### XTNES error estimation

The single-voxel electron density and vanadium concentration uncertainties, at 52-nm voxel size, were estimated on the basis of their standard deviations (SDs) (σ). Measurement uncertainty of electron density was calculated on a region of air surrounding the imaged catalyst pillars and found to be 0.007 n_e_ Å^−3^ for the pristine sample and 0.006 n_e_ Å^−3^ for the used catalyst sample. The measurement uncertainty of the vanadium concentration was calculated similarly and found to be 0.0042 n_V_Å^−3^ for the pristine catalyst and 0.0034 n_V_Å^−3^ for the used catalyst. Oxidation state and pre-peak intensity are undefined in the air region, so a similar approach cannot be used. To estimate those errors, we selected an isolated micrometer-sized grain from the used sample, which shows a single chemical composition, (VO)_2_P_2_O_7_, and calculated the SD of edge center energy and pre-peak intensity in that grain. This way, we determined the measurement uncertainty of edge center energy to be 1.1 eV, or 0.22 eV in oxidation state. The uncertainty of pre-peak intensity was found to be 0.02 (1/n_e_Å^−3^). These uncertainty values are used as estimate for both samples because of the highly heterogeneous structure of the pristine catalyst.

When calculating the mean value of a region of interest, such as the surface distance maps shown in Fig. 3C, the error is calculated using the standard error of the mean (SEM), which equals the SD of the voxels included in the SD calculation divided by square root of the number of voxels. The error values were too small to be visible as error bars in Fig. 3C.

## SUPPLEMENTARY MATERIALS

Supplementary material for this article is available at http://advances.sciencemag.org/cgi/content/full/7/24/eabf6971/DC1
